# Multi-Objective Optimization in Single-Shot Drilling of CFRP/Al Stacks Using Customized Twist Drill

**DOI:** 10.3390/ma15051981

**Published:** 2022-03-07

**Authors:** Muhammad Hafiz Hassan, Jamaluddin Abdullah, Gérald Franz

**Affiliations:** 1 School of Mechanical Engineering, Universiti Sains Malaysia, Nibong Tebal 14300, Malaysia; mhafizhassan@usm.my; 2 Laboratoire des Technologies Innovantes, UR UPJV 3899, Avenue des Facultés, Le Bailly, 80025 Amiens, France

**Keywords:** CFRP/Al7075-T6, thrust force, twist drill, single shot

## Abstract

In recent years, the use of CFRP with titanium and/or aluminum to form materials for stacking has gained popularity for aircraft construction. In practice, single-shot drilling is used to create perfectly aligned holes for the composite-metal stack. Usually, standard twist drills, which are commonly available from tool suppliers, are used for practical reasons. However, existing twist drill bits exhibit rapid wear upon the drilling of composite-metal stack layers in single shot, due to the widely contrasting properties of the composite-metal stack, which causes poor surface quality. The stringent quality requirements for aircraft component manufacturing demands frequent drill bit replacement and thus incurs additional costs, a concern still unresolved for aircraft component manufacturers. Owing to highly contrasting properties of a composite-metal stack, it is obvious that standard twist drill cannot fulfil the rigorous drilling requirements, as it is pushed to the limit for the fabrication of high-quality, defect-free holes. In this work, customised twist drills of a tungsten carbide (WC) material with different geometric features were specially fabricated and tested. Twenty drill bits with customised geometries of varying chisel edge angle (30–45°), primary clearance angle (6–8°), and point angle (130–140°) were fabricated. The stacked-up materials used in this study was CFRP and aluminum alloy 7075-T6 (Al7075-T6) with a total thickness of 3.587 mm. This study aims to investigate the effect of twist drill geometry on hole quality using drilling thrust force signature as indicator. All drilling experiments were performed at spindle speed of 2600 rev/min and feed rate of 0.05 mm/rev. Design of experiments utilising response surface methodology (RSM) method was used to construct the experimental array. Analysis of variance (ANOVA) was used to study the effect of parameters and their significance to the thrust force and thus the hole quality. The study shows that the most significant parameter affecting the drilling thrust force and hole surface roughness is primary clearance angle, followed by chisel edge angle. Correlation models of CFRP thrust force (*Y*_1_), Al7075-T6 thrust force (*Y*_2_), CFRP hole surface roughness (*Y*_3_), Al7075-T6 hole surface roughness (*Y*_4_) as a function of the tool geometry were established. The results indicated that the proposed correlation models could be used to predict the performance indicators within the limit of factors investigated. The optimum twist drill geometry was established at 45° of chisel edge angle, 7° of primary clearance angle, and 130° of point angle for the drilling of CFRP/Al7075-T6 stack material in a single-shot process. The error between the predicted and actual experiment values was between 6.64% and 8.17% for the optimum drill geometry. The results from this work contribute new knowledge to drilling thrust force signature and hole quality in the single-shot drilling of composite-metal stacks and, specifically, could be used as a practical guideline for the single-shot drilling of CFRP/Al7075-T6 stack for aircraft manufacturing.

## 1. Introduction

Carbon fiber-reinforced polymer (CFRP) composites are commonly used in commercial aircraft, such as Airbus A350 and Boeing 787, as elements of the wing structure, the fuselage, and other secondary components due to its high strength to weight ratio compared to other metallic parts. Composites are used in parts of the aircraft that encapsulate the metal structure of the material and function as a skin. The composite part is attached to the metal structure by producing holes in a single-shot drilling process where the rivet or fastener is installed to the stack to complete the assembly process. In recent years, the use of CFRP with titanium and/or aluminum to form materials for stacking has gained popularity. In general, this is true in terms of usage in systems such as aerospace structures, which are prone to dangerous mechanical loads [1]. The relation of these materials usually requires the manufacturing of high-quality attachment holes. In actuality, the holes for critical components, such as the wing and tail planes, are created via a series of routines that involves pre-drilling of each surface followed by a deburring system to complete the high-performance cycle [2]. The stacking material is then arranged and positioned (mechanically) properly before the hole reaming process. The difference in structural features (e.g., specific elastic components, coefficient of thermal expansion, etc.) frequently causes difficulties in achieving the tolerances required. 

Single-shot drilling of CFRP/metal is the preferred technique for minimising positioning errors and process time [3]. Problems with drilling these dissimilar materials usually include severe tool wear, heat-induced damage, oversized drilled holes, roundness deviation, and metal burr formation [4,5,6]. Available drill bits from the standard catalogue cannot fulfil the conflicting requirements due to the different properties of the materials. Drilling with a twist drill design is not recommended for composite panels as this type of drill bit will contribute to a higher probability of delamination after the drilling process [7,8,9]. When drilling the CFRP panel with a metal stack, the most suitable drill geometry, which is a compromise of the needs of each material, must be applied to optimally drill the stack material, especially during a single-shot drilling process. In order to guarantee the machining quality and the surface integrity, the selection of both the optimal cutting parameters, namely feed rate and spindle speed, is required, as well as appropriate drill geometries such as drill diameter, chisel edge angle, primary clearance angle, or point angle.

The quality of the assembly of hybrid structure is conditioned by CFRP delamination, which is strongly related to feed rate [10,11] and, to a limited extent, to spindle speed [12]. In order to limit the occurrence of delamination in laminated composites, it is recommended to perform the drilling with low feed, smallest drill point angle, and high spindle speed [13,14]. Furthermore, Zitoune et al. [15] found that one of the major problems arising in aluminum is built-up edge (BUE), which can be eliminated by increasing the spindle speed. Unfortunately, these cutting conditions result to longer processing times (usually several minutes per hole) and lead to long chips in aluminum alloy when drilling CFRP-Al stacks. The production of non-fragmented chips generates a degradation of hole surface roughness. They also discovered that thrust force and chip breakability were significantly impacted by drill diameter and feed rate during drilling of the CFRP/aluminum stack, while the effect of spindle speed could be neglected. In this study, various drill diameters (4–8 mm) of plain carbide drills (K20) with a typical point angle of 118° have been used to investigate the influence of spindle speed (1050–2750 rev/min) and feed rate (0.05–0.15 mm/rev) on thrust force and hole surface quality. A spindle speed of 2020 rev/min and a feed rate above 0.1 mm/rev were found to obtain better hole surface roughness in aluminum panel, due to the formation of small, well-broken chips during the drilling operation. Concerning the CFRP, similar surface roughness values (2–4 µm) have been found at low feed rates (f = 0.05 mm/rev), regardless of spindle speed or drill diameter. Whereas it was found that surface roughness decreased with increasing feed rate for all drill diameters, the influence of spindle speed was found to be insignificant. Moreover, it was established that the shape and size of the chips were strongly influenced by the choice of the feed rate [16]. Similarly, Zhang et al. [17] investigated the influence of cutting parameters, such as spindle speed (2700–5900 rev/min) and feed rate (0.03–0.07 mm/rev), and drill bits on thrust force and hole quality of CFRP/aluminum stack. They concluded that a spindle speed of 4000 rev/min with a feed rate of 0.04 mm/rev constituted the optimum process parameters to achieve the hole surface roughness required by industry (CFRP: Ra ≤ 3.2 μm, Al: Ra ≤ 1.6 μm) when drilling CFRP/aluminum stacks with a 5 mm diameter CVD diamond-coated WC twist drill with a shorter chisel edge, two major cutting edges and a point angle of 90°.

In the single-shot drilling of a CFRP/metal stack, the choice of an appropriate drill tool geometry represents a major challenge since it greatly involves hole surface integrity. According to Rubio et al. [18], tool design influences the amount of delamination and thrust force for drilling CFRP. Durao et al. [19] performed a comparative study with five different drill point geometries (twist drills with 120° and 85° point angles, Brad type, Dagger type, and customised step type) and feed rate (0.02–0.12 mm/rev) on thrust force, delamination, and hole surface roughness. In accordance with the aforementioned previous studies, it was found that the thrust force increased sharply with increasing feed rate during drilling, regardless of drill geometries. In contrast, no relevant conclusion concerning the influence of feed rate on hole surface roughness could be given as the results were too scattered. Authors finally reported that a 120° twist drill should be used for minimal delamination and that a step drill could act as a good alternative, especially to reduce thrust forces. Krishnaraj et al. [20] proposed that the most common drill point angle is at 118° to achieve minimum thrust force and torque formation for CFRP material. However, the range of drill point angle depending on the machined materials may vary between 80° and 140°. Various research studies [21,22,23,24] dealing with the effects of drill bit geometry in the drilling of CFRP/metal stacks have shown that a drill bit with double point angle performed soundly in terms of tool wear, thrust force, and surface quality of the hole compared with a twist drill bit. El Bouami et al. [25] focused on the effect of interaction of cutting parameters and tool geometry on cutting force and delamination in drilling unidirectional CFRP/Al stacks using design of experiments and the Taguchi approach. Drilling was conducted using carbide twist drills with three different point angle value in the range from 120° to 150°. This study has shown that the drilling of CFRP/aluminum stacks required sufficiently high feed rates to produce fragmented chips and thus to obtain better hole quality in aluminum panels. However, a higher feed rate also implies a degradation of CFRP hole quality. Both point angle and feed rates have been found to be the predominant factors on thrust forces in CFRP panels, while only point angle has a significant influence on thrust force in aluminum panels. In a subsequent work, El Bouami et al. [26] also studied the influence of cutting parameters and tool geometry (twist drill, step drill, and point spur drill) on hole wall damage when drilling unidirectional CFRP/Al stacks. Results have shown that spur drill provide better drilling performances than other tested geometries by causing small damage extension in the hole perimeter. However, rapid tool wear was observed with increasing feed rate. Moreover, the step drill exhibited lower thrust forces compared to those performed with both twist and spur drill. They also observed that the drilled hole surface quality was directly linked to the drilling parameters, and concluded that low feed rates coupled with optimal coated tool geometry could provide good results in terms of thrust force reduction and delamination phenomenon minimisation. Feito et al. [4] concluded that using a twist drill with a low point angle in the range of 90–108° is recommended for reducing delamination in CFRPs. The helix angle also contributes to reducing cutting forces. The most common helix angles range between 12° and 38° depending on the application [27]. Sui et al. [28] proved the performance of the double-cone integrated tool in a single-shot drilling process on metal-to-metal stack laminates. They found that a double-cone integrated drill reduces the thrust force when compared with a standard twist drill chosen as reference, due to its longer cutting edge. An increased feed rate was prone to produce smaller chips. The effect of adding an aluminum layer on the CFRP’s hole quality during a one-shot drilling process using a standard twist drill was investigated by Madhi et al. [29]. The influence of the drilling sequence (CFRP/Al or Al/CFRP) was also reported in this study. From their research, cutting speed had a dominant influence on surface damage. The hole damage observed on aluminum and composite panels, such as feed mark, matrix smearing, delamination, and surface cavities have been further investigated. Jia et al. [30] proposed a new twist drill design called a multi-margin structure, which is located at the drill bit step. The bit could improve the thrust force and delamination by about four times compared with the standard twist drill bit. However, the design proposed is complicated and costly due to more modification need to be designed at the cutting point and the body. However, the correlation between the tool geometry and drilling parameters affecting the drilling force and hole surface roughness have not yet been discovered.

Fewer studies have investigated the optimisation of the standard twist drill design in a single-shot drilling process of CFRP/Al stack materials. As CFRP/Al stacks are widely used in aerospace applications, especially on commercial aircraft, it is necessary to study the drill performance for this kind of stack. Hitherto, no attempt has been made to design customise twist drill tool geometry, which is more economical compared to previous drill design, for drilling stack material in a single-shot drilling process. This study aims to design a unique twist drill geometry that fulfils drilling requirements for CFRP/Al7075-T6 stack materials. This geometry is not available in the standard catalogue from drill manufacturers and the twist drill geometry is used for aircraft assembly process. Thus, a systematic experimental design based on response surface methodology (RSM) is introduced in this study to determine the explicit effect of drill geometry parameters involved in the drilling of CFRP/Al7075-T6. In brief, the used RSM includes regression analysis and statistical design of experiments for constructing the global optimisation of the testing parameters [31]. It is also one of the most widely used methods for solving optimisation problems in manufacturing environments [32,33,34]. The thrust force and hole surface roughness will be taken into consideration, with their interactions quantified using numerical modeling techniques.

## 2. Materials and Methods

### 2.1. Materials

The stack materials used in this study were CFRP and aluminum alloy 7075-T6 (Al7075-T6), with a density of 1.601 g/cm^3^ and 2.597 g/cm^3^, respectively. The CFRP composite specimen consists of 26 unidirectional plies of 0.125 mm each in thickness, for a total laminate thickness of 3.25 mm. The 26 unidirectional plies were made of carbon/epoxy prepreg manufactured by Hexcel Composite Company. The stacking sequence was [45/135/90_2_/0/90/0/90/0/135/45_2_/135] s. A 0.08 mm thin layer of glass/epoxy woven fabrics was then used at the top and bottom of the CFRP laminate to avoid delamination at the entrance and exit of the hole during drilling. Thus, the final thickness of the whole composite panel, including the paint application, was 3.587 mm. 

Throughout the curing process, the CFRP was compacted using a vacuum pump under controlled atmospheric conditions. A mold for the laminate was prepared and placed inside the autoclave. The cure cycle consisted of increasing the temperature to 180 °C at a rate of 3 °C/min, which was maintained for 120 min. Then, the temperature was returned room temperature at the same rate. The whole cycle was conducted at pressure of 700 kPa in an autoclave and placed in a vacuum bagging, which was evacuated to 70 kPa [35]. Hence, by applying that curing recipe, the nominal fiber volume fraction was 60%. 

Table 1 summarises the mechanical and physical properties of the stack materials used in this work.

### 2.2. Cutting Tool Fabrication

The drill bit type is a combined drill and countersink. The diameter of the drill is 4.826 mm, and the countersink is 10 mm. Sintered rod of tungsten carbide was chosen as the material for the drill bit due to high resistance to wear during drilling of abrasive materials such as CFRP. The composition of the tungsten carbide (WC) rod was WC ~93.36 wt % and Cobalt ~6.64 wt %. It has a density of 14.35 g/cm^3^ and hardness value of 1625 HV, both of which are significantly higher than the workpiece material. The drilling tools can easily shear the surface of workpiece material without causing breakage of the tool itself. 

**Figure 1:**
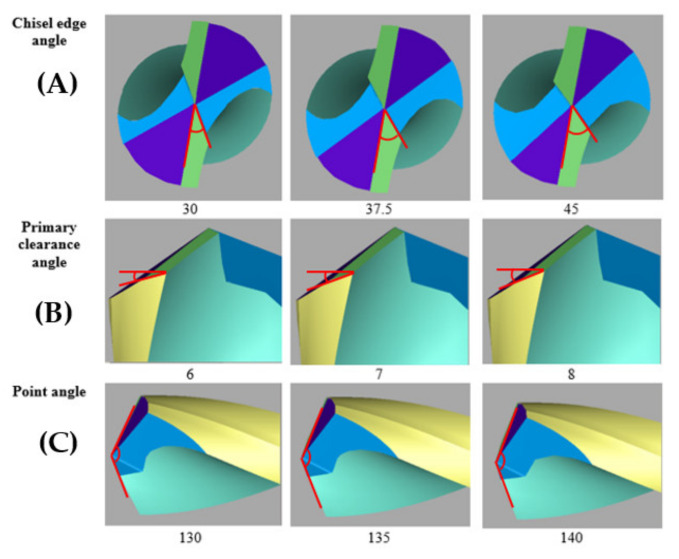
Details of tool geometry with minimum and maximum range.

**Figure 2:**
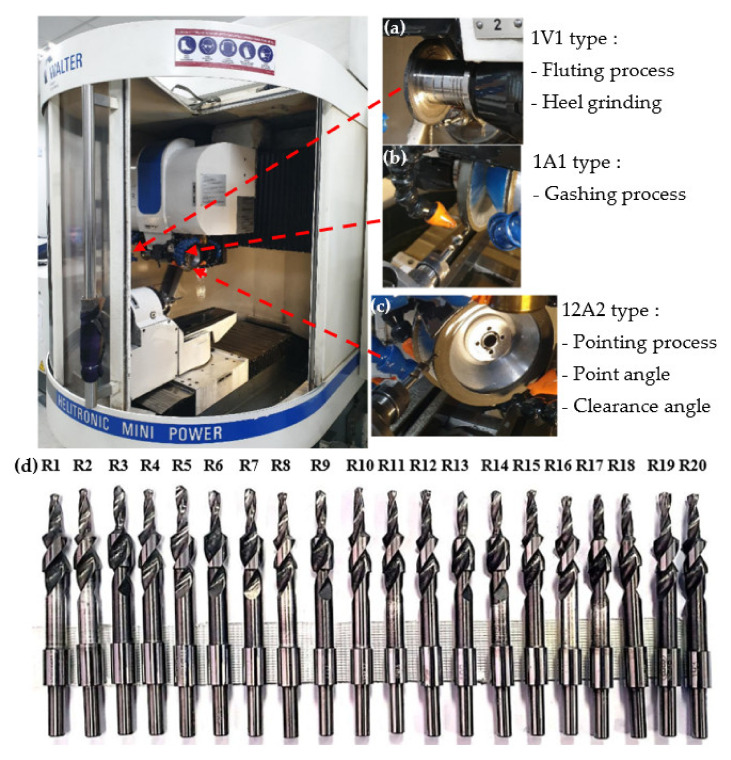
Location of grinding wheel for the tool fabrication (**a**) fluting wheel, (**b**) gashing wheel, (**c**) clearance/point angle wheel, (**d**) finished drill with customised geometry of twist drill types.

Figure 1 illustrates the drills with specially designed custom drill geometry designed using Helitronic Tool Studio version 1.9.216.0 (Walter Maschinenbau GmbH, Garbsen, Germany). The grinding of a cutting tool requires a specific wheel to follow several sequential operation steps, which started with a pointing process, followed by gashing and clearance. For the gashing process, the chisel edge angle of 30° to 45° was set in the program, meanwhile in the clearance process the primary clearance angle was set from 6° to 8°. Finally, in the pointing process, the point angle was set at 130° to 140°. Figure 2a–c illustrates the process of manufacturing and the wheel type uses in customising the twist drill design by using the CNC grinding machine (Walter Maschinenbau GmbH, Garbsen, Germany). Figure 2d shows a full set of finished custom-made standard twist drill with variation of the three aforementioned angles, with the values summarised in Table 2. The experimental procedure was designed using design of experiment (DOE). DOE is a common method to construct the number of experiments to determine the relationship between input and output of independent variables based on a statistical approach. The twenty trials given in Table 2 were developed from RSM using a central composite design (CCD), as explained in detail in Section 2.5.

### 2.3. Drilling Process and Thrust Force Measurement

**Figure 3:**
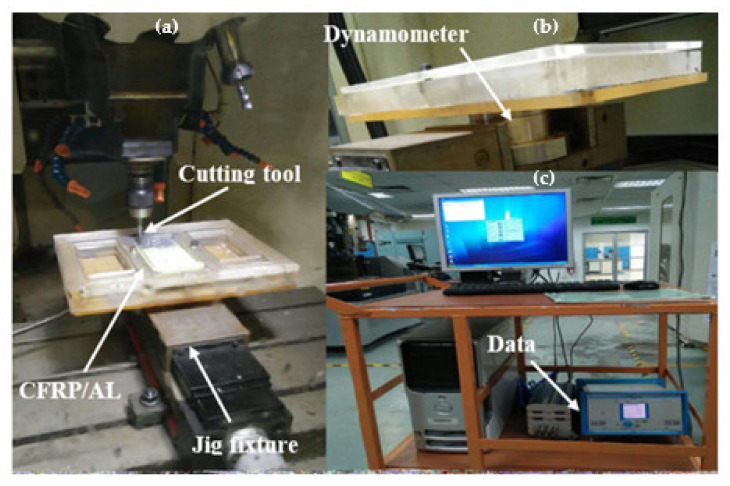
(**a**) Actual set-up for drilling process; (**b**) position of dynamometer; and (**c**) amplifier and computer data acquisition of thrust force measurement.

To optimise the drill bit geometry, the drilling process of a stack material was performed using a computer numerical control (CNC) machine Fanuc Robodrill α-T21iFLb model, which has a variable spindle speed up to 10,000 rev/min with spindle drive motor of 3.7 kW at a continuous rating. The range of feed rate is within 1 to 30 mm/min for a standard rate and 48 m/min (*x*-axis, *y*-axis, *z*-axis) for a rapid transverse rate. The drilling process was executed in a single-shot process, which starts from the CFRP to the Al7075-T6 panel. The type of CNC used and the detailed set up of the workpiece for the drilling process are illustrated in Figure 3. The stack panels were slot in and clamped inside the fixture during the drilling process. In this study, a spindle speed of 2600 rev/min and a feed rate of 0.05 mm/rev were used for all runs to observe the significant effect of the customised twist drill geometry. Dry drilling condition is used in this experiment to mimic the actual drilling process during manufacturing of the panel. Although the use of cutting fluids can enhance the machining efficiency and improve the tool life by dissipating the heat produced at the cutting region, the cyclic utilisation of coolant in composite-metal stack drilling processes is uneconomic and environmentally unfriendly due to the heavy pollution of the powdery CFRP’s chips, justifying the interest in reducing its use. Furthermore, the reduction of the amount of cutting fluid used is also interesting to avoid cleaning operations after drilling. In CFRP/metal stack drilling processes, especially when the metallic panel is titanium, minimum quantity lubrication (MQL) can be used to limit the adhesion of metal on the cutting edge, delay tool wear, and reduce the high temperatures produced during Ti drilling. However, Seo et al. [37] indicated that MQL machining induced an increase in cutting force, and thus delamination, due to the reduced softening of the material induced by the reduced cutting temperatures. Fernández-Pérez et al. [38] investigated the influence of the MQL level on the hole quality, tool wear, and power consumption during the drilling of Ti/CFRP/Ti stacks with a diamond-coated carbide drill bit of 7.6 mm in diameter. Results have shown that the use of MQL significantly affected the behavior of the process during drilling the Ti layers while no impact was observed on the performance of the composite layer drilling.

To acquire the thrust force signature, a dynamometer is attached to the worktable of the CNC machine. When a force is detected during the drilling operation, the test data will be transmitted to the data acquisition system. Then, the detected signal will be amplified, and the output will be displayed in the computer in the form of a thrust force signature versus cutting time. A dynamometer (Kistler 4 component dynamometer type 9272), as shown in Figure 3a,b, was used to monitor the thrust force during the drilling process of the stack material. The workpiece that was clamped by the jig was mounted on the dynamometer on the table of the milling machine.

The data acquisition system, which was connected to the dynamometer, consists of a multichannel charge amplifier (type 5070) and Kistler DynoWare software (IMC Measurement and Control Version 3.2 Rev 2) The thrust force signature was generated when the dynamometer consisting of a four-components sensor transfers the charge signal to the multichannel charge amplifier. The multichannel charge amplifier converts the resulting charge signal, which was proportional to the applied force, to voltage. The resulting signals were converted to force by the calibrated data and displayed in the software.

### 2.4. Hole Surface Roughness Measurement

It is widely accepted that hole surface roughness can be considered as one of the main outputs used to evaluate the drilling performance of composites [39,40,41]. This characteristic is measured by monitoring the irregularities of the surface of the workpiece. Drilling parameters and drill bit geometry sharply affect hole surface roughness [42,43]. For a composite panel, to ensure a good quality of hole surface roughness after drilling process, the wall of the drilled hole must be free from any surface defects (epoxy burn, delamination, and void); while for the aluminum panel, there should be a shiny surface on the hole. Based on previous research [20,28,29,30], it is a challenge to minimise the hole surface roughness of the composite part due to its inhomogeneous properties compared to the metal part. Moreover, CFRP’s roughness should be cautiously construed due to the lower measurement reliability in composites compared to one acquired in metals [2]. According to aircraft manufacturer’s specification standards, the cut-off wavelength should be 0.8 mm and the evaluation length for measurements in thickness direction should be 1.6 mm for range of thickness between 3.2 mm to 6.0 mm.

**Figure 4:**
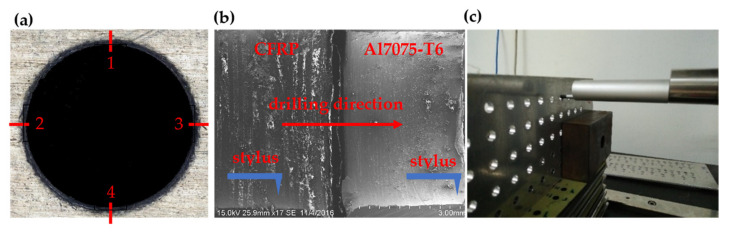
Surface roughness analysis: (**a**) position of measurement, (**b**) stylus location during measurement, (**c**) workpiece position during measurement.

Figure 4a shows the detailed location and type of probe, which is a skidless type, that were used for hole surface roughness measurement. The stylus profiler with a diamond tip of 0.2 μm radius, attached to a delicately balanced arm, is dragged along the hole surface (Figure 4b). As the diamond tip encounters peaks and valleys, the tip will be raised and lowered. Subsequently, the vertical motion of the stylus is detected electrically. The electrical signals undergo amplification and a digital conversion process. The result from the stylus measurement is then expressed as a single parameter (R_a_). The arithmetic mean roughness R_a_ represents the arithmetic average of the ordinates’ absolute values of the measured profile along to the midline in a sampling length. In other words, R_a_ indicates the average difference between peaks and valleys of the roughness profile. This roughness parameter is widely used in industry to assess the hole quality in the drilling of CFRP material. During the setup, the workpiece is placed perpendicularly to the stylus, with the aid of dummy blocks, as shown in Figure 4c.

### 2.5. Response Surface Methodology (RSM)

RSM is an essential technique for solving robust design problems and to optimise a product or process; it has many advantages compared to Taguchi’s method [44]. RSM has been employed to optimise the twist drill geometry for drilling a stack material in a single-shot drilling process. The interactive relationship of the input variables was investigated in this optimisation study. The design of the RSM was performed using a central composite design (CCD), which is the most popular of all second-order designs. The CCD comprises a full factorial design (2k) with 2k of axial or star points and centre points, where k is the number of factors [45]. The factors are varied over three levels between −1 and +1. In this study, the chosen factors and their coded levels are presented in Table 3. The numerical experiment runs were generated according to the equation CCD = 2*^k^* + 2*k* + 6, where k is the number of factors with replications at the design centre. For optimisation, a quadratic model was used to fit and estimate the minimum point. These points were used to develop the mathematical model for each response, as shown in Equation (1) [46,47]:

(1) $$Y=\beta_{0}+\overset{k}{\underset{i}{\sum }}\beta_{i}X_{i}+\overset{k}{\underset{i=1}{\sum }}\beta_{ii}X_{i}^{2}+\overset{k}{\underset{i=1}{\sum }}\overset{k}{\underset{j=1}{\sum }}\beta_{ij}X_{i}X_{j}$$

where *Y* denotes the predicted response, *β*_0_ represents an offset term, *X_i_* et *X_j_* are the input variables, *β_i_* is the *i-th* linear coefficient, and *β_ij_* the *ij-th* interaction coefficient.

## 3. Results and Discussion

### 3.1. Thrust Force Analysis

**Figure 5:**
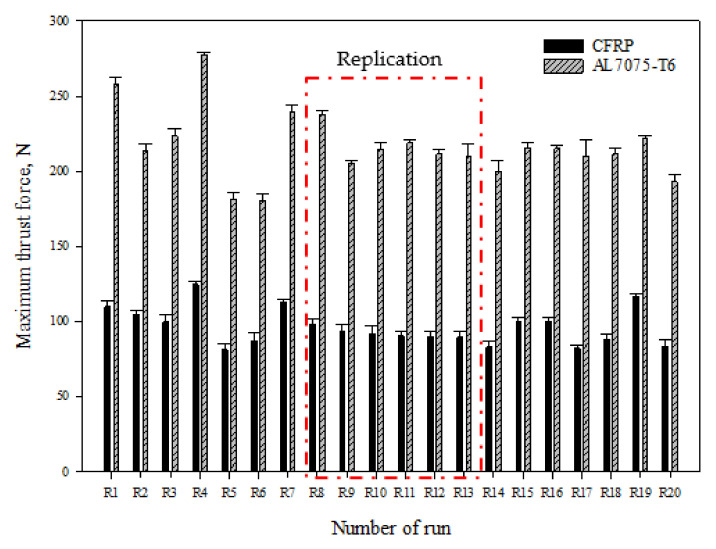
Average measurement of maximum thrust force for CFRP/Al7075-T6 stack materials in the RSM test.

Figure 5 presents the maximum thrust forces (*F_tmax_*) measured at CFRP panel, ranging from 81.16 N to 124.56 N as the range of values in all of the RSM tests. The smallest *F_tmax_* (81.16 N) was recorded at R5, with a cutting geometry of 30° chisel edge angle, 8° primary clearance, and 140° point angle. The larger *F_tmax_* (124.56 N) was recorded at R4 (30° chisel edge, 6° primary clearance angle, and 140° point angle). 

The drill advancing into Al7075-T6 panel produced similar thrust force, ranging from 180.674 N to 223.574 N, as the range of values obtained from five holes. The largest *F_tmax_* was recorded at R4, the same as the CFRP panel, while the smallest *F_tmax_* was recorded at R6 with a cutting geometry of 37.5° chisel edge angle, 7° primary clearance angle, and 130° point angle. 

The results obtained from replication tools are consistent since the standard deviation value is about 3.05 N from the mean value of the experiment, which confirms that the consistency of the manufacturing tool is acceptable.

#### 3.1.1. Regression Model and Analysis of Variance (ANOVA) for the Maximum Thrust Force

The regression models for the responses were selected based on the highest order polynomials suggested by the Design Expert 7.0 software (Stat-Ease, Inc., Minneapolis, MN, USA). The best fit was obtained by selecting a quadratic model for the minimum thrust force of stack materials. The *p*-value of the model, which is calculated from ANOVA, must not exceed 0.05, since a 95% confidence level is selected for the analysis.

The final empirical models are given in terms of actual factors A, B, and C (cf. Table 3) for the maximum thrust force for the CFRP panel (*Y*_1_):

(2) $$Y_{1}=-322.93212-12.78427A+71.30139B+6.42949C+0.0897AC-1.3362BC+6.86894B^{2}$$

In the same manner, the maximum thrust force for the Al7075-T6 panel (*Y*_2_) is given as below:

(3) $$Y_{2}=-791.496-13.8033A+163.4374B+12.427C+1.7756AB-1.8621BC$$

Table 4 and Table 5 depict the summary of the analysis of variance (ANOVA) for each regression model. The variable of interest in this statistical analysis is the coefficient of the determination (*R*^2^), a statistical measure used to determine on how well the regression line fits the actual data points. The higher the *R*^2^ value, the better the model is at making predictions about the system. The information from ANOVA indicates that the *R*^2^ values are 0.934 and 0.897 for responses *Y*_1_ and *Y*_2_, respectively, proving that the chosen quadratic models are good response predictors. Furthermore, the probability values (*p*-values) are less than 0.05, which indicates that the designed model is significant. In addition, small lack of fit and residual values were recorded for each model, indicating that the regression model sufficiently fits the experiment data. It should be noted that the pure error was registered at the value of zero, since the repeated runs generated minimum error for the experimental work. The small coefficient of variation (C.V.) value indicated that the experimental data were precisely dispersed around the mean value. 

Table 4 indicates that the primary clearance angle (B) (percentage of contribution (PC): 58.78%) has a far greater effect on the maximum thrust force for CFRP than the chisel edge angle (A) (PC: 8.87%) or point angle (PC: 1.71%). Furthermore, the contribution on this output of the interaction between the primary clearance angle and the point angle is stronger (PC: 12.57%) than that between the chisel edge angle and point angle (PC: 3.19%).

Based on ANOVA results shown in Table 5, primary clearance angle (B) significantly influences the maximum thrust force value for Al7075-T6 (PC: 52.34%), while point angle (C) has no effect. Moreover, the interactions of the two input variables, namely AB and BC, were observed in this response and it was found that the interaction of the primary clearance angle with the chisel edge angle (PC: 16.29%) was higher than with the point angle (PC: 7.96%). 

**Figure 6:**
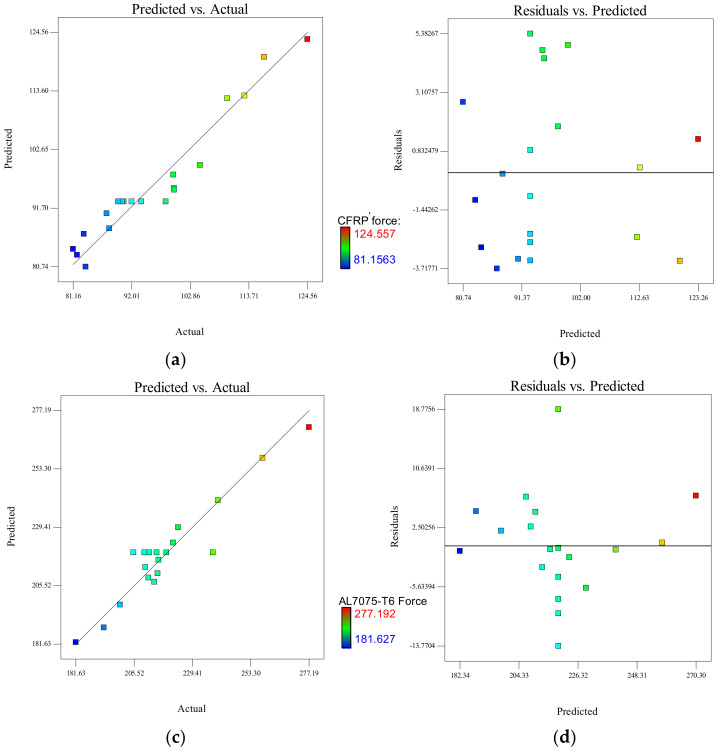
CFRP maximum thrust force analysis for (**a**) actual and predicted plot and (**b**) predicted and residual plot; Al7075-T6 maximum thrust force analysis for (**c**) actual and predicted plot and (**d**) predicted and residual plot.

The predicted central composite design for CFRP results is plotted against actual results in Figure 6a. This graph helps to detect values that are not easily predicted by the regression model. The plots below demonstrate that each model provides a good fit; hence, the predicted and obtained values from the generated model are in agreement with the actual experimental results. 

For Al7075-T6 panel, the actual and the predicted values for *F_tmax_* are presented in Figure 6c. It is clearly visible from the graph that most of the values of *F_tmax_* fall in the proximity of the centreline, which indicates the model is fitted in better terms. The actual and predicted models were the important parts that determined *F_tmax_* for the CFRP and Al7075-T6 stack materials. 

Figure 6b,d shows the residuals versus predicted *F_tmax_* for both materials. It signifies that for all values of the response in terms of the scatter plot, the variance of the original observation obtained remains constant, which is an indication that there is no need for transformation of the response variables. The RSM model is significant and can be utilised to predict the response since two-thirds of the data points are within the standard error estimation (SEE), which is above or below the least squares line for a data set with a normal linear relationship [48]. From Figure 6b,d the SEE values of *F_tmax_* for CFRP and Al7075-T6 are 3.05 N (i.e., if the predicted value is 102.45 N, the actual value is in the range from 99.4 N to 105.5 N) and 6.86 N (i.e., if the predicted value is 220.45 N, the actual value is in the range from 213.59 N to 227.31 N), respectively.

#### 3.1.2. Effect of Geometry Parameters on Maximum Thrust Force (CFRP/Al7075-T6)

**Figure 7:**
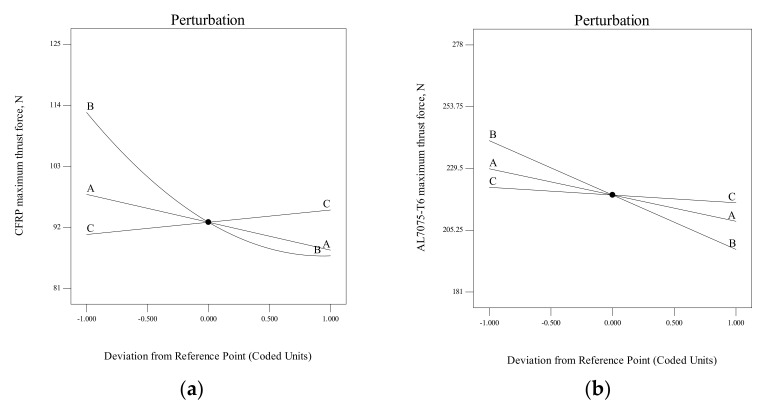
Perturbation plot for maximum thrust force for (**a**) CFRP and (**b**) Al7075-T6 (Note: A = Chisel edge angle, B = Primary clearance angle, C = Point angle).

The perturbation plot was utilised to examine the sensitivity of the independent variables (factors) to the responses of *Y_1_* and *Y_2_*, as presented in Figure 7. It is apparent that both the maximum thrust force for CFRP and Al7075-T6 materials are affected by the primary clearance angle (B) during the drilling of stack material process. The negative slope of B indicates that the *F_tmax_* for both materials decrease with increasing primary clearance angle. Same trends are observed with the chisel edge angle (A) where the *F_tmax_* for both materials decreases with increasing chisel edge angle. For the point angle (C), the *F_tmax_* for CFRP panel slightly increases when point angle increases from 130° (lower level −1) to 140° (higher level +1). However, for Al7075-T6 panel, point angle shows a modest influence on the *F_tmax_* in this study since its variation from 130° to 140° results in insignificant changes in *F_tmax_*.

**Figure 8:**
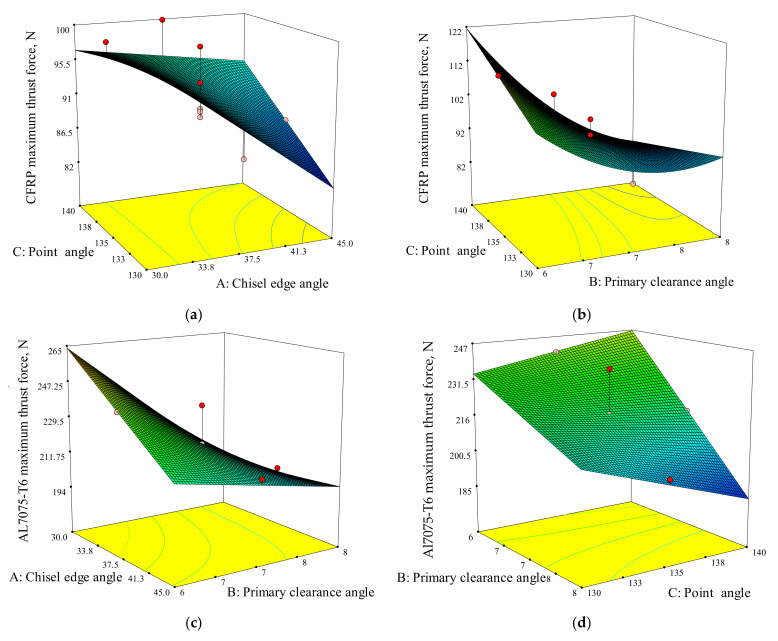
3D response surface for maximum thrust force for CFRP: (**a**) chisel edge angle and point angle, (**b**) primary clearance angle and point angle. 3D response surface for maximum thrust force for Al7075-T6: (**c**) chisel edge angle and primary clearance angle, (**d**) point angle and primary clearance angle.

Figure 8 illustrates the 3D response surface and contour plots of the quadratic and linear models for CFRP (*Y*_1_) and Al7075-T6 (*Y*_2_). The interactive relationship between two of the most significant factors was determined based on the 3D response surface. In this case, the selection is according to the level of sensitivity towards the responses, as plotted in the perturbation plots (Figure 7). 

In Figure 8a,b, there are two interactions shown that influence the *F_tmax_* for CFRP panel. The results show that the primary clearance angle (B) and point angle (C) is the first interaction and the chisel edge angle (A) and point angle (C) is the second interaction where the *F_tmax_* for CFRP (*Y*_1_) values are varied. Notably, other variables were set as constant for the reference point. As shown in Figure 8a, the smallest *F_tmax_* for CFRP is identified at 130° point angle and 45° chisel edge angle. From Figure 8b, it is shown that the smallest *F_tmax_* value is observed for 140° of point angle and 8° of primary clearance angle. The lower point angle (130°) will result in lower thrust force due to the non-homogenous behaviour of the CFRP. The point angle is a critical factor since it is the first contact point that cuts the fibres and matrix at the drilled hole wall [49]. It is more effective to cut the CFRP, which is stacked layer by layer, at a more acute angle (130°) because the cutting area is smaller, which minimises the delamination damage [50].

Figure 8c shows the interaction between the chisel edge angle (A) and the primary clearance angle (B) that influences *F_tmax_* for Al7075-T6 panel while Figure 8d shows the interaction between primary angle clearance (B) and point angle (C). The smallest *F_tmax_* for drilling Al7075-T6 is obtained at 8° of primary clearance angle and 30° of chisel edge angle, as shown in Figure 8c, and 140° of point angle and 8° of primary clearance angle, as shown in Figure 8d. Concerning the Al7075-T6 material, a higher point angle will lead to lower thrust force at constant drilling parameters, due to the decrease in tool–chip contact area. Therefore, the penetration resistance of the cutting tool during drilling process must be reduced [51].

### 3.2. Hole Surface Roughness Analysis

**Figure 9:**
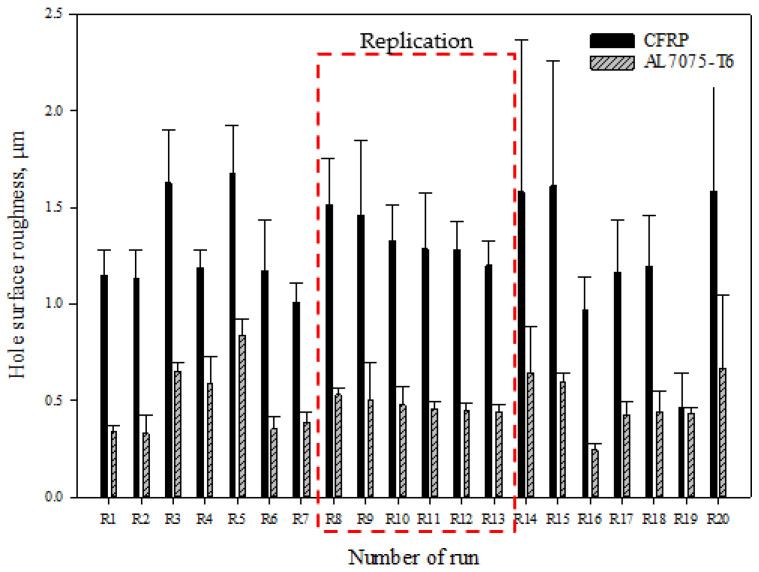
Measured results of average hole surface roughness for the stack material in extended study.

According to Figure 9, the average surface roughness value of stack material hole varied from 0.4649 µm to 1.6794 µm for CFRP and 0.2423 µm to 0.8343 µm for the Al7075-T6 panel. Variations indicate that the effects of the tool’s geometrical features on the response (surface roughness) are significant. Moreover, the variations in hole surface roughness for CFRP are higher compared to those obtained in Al7075-T6 material because of the inhomogeneous nature of the laminates, which is designed ply by ply with different stacking sequences [52]. From these results, it can be stated that the combination of drill design R19 (45° chisel edge angle, 6° primary clearance angle, and 140° point angle) produces the lowest surface roughness of the CFRP panel with a value of 0.4649 µm, while R16 (45° chisel edge angle, 6° primary clearance angle, and 130° point angle) produces the lowest surface roughness for Al7075-T6, with a value of 0.2423 µm. That shows that the optimal combination of drill bit geometry can produce smooth surface roughness and, for this case, it provides a better shearing action during the drilling process.

**Figure 10:**
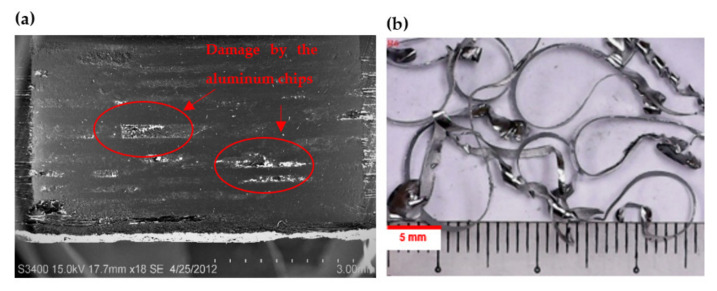
(**a**) Cross-section of CFRP hole for surface roughness measurement; (**b**) continuous aluminium chips produced by the bit design.

Meanwhile, the combination of drill bit design R5 (30° chisel edge angle, 8° primary clearance, and 140° point angle) produces the highest hole surface roughness for both the CFRP and Al7075-T6 panels, with values of 1.6794 µm and 0.8343 µm, respectively. The main reason for the highest hole surface roughness value for both materials obtained from the combination of this drill bits is the lower wedge angle provided during the cutting process. Increasing the primary clearance angle relative to the wedge angle of the tool will reduce the cutting efficiency. In drilling the CFRP panel, a larger wedge angle is needed to improve the cutting mechanism as the laminate has various fibre orientations. The bent fibres due to the cutting process tend to bounce back after the cutting edge passes, creating fuzziness while drilling with lower wedge angle [53]. The average result obtained for the replication tools regarding the Al7075-T6 hole surface roughness is consistent since the standard deviation value is about 0.05 µm. This standard deviation values are obtained from the mean value of the experiment, which proves the consistency of tool manufacturing is acceptable. However, for CFRP panels, the high dispersion in hole surface roughness for the replication runs (R8-R13) is due to the chip clogging at the flute [54]. Long and continuous stringy chips tend to wrap around the cutting tool, which may become an issue for continuous an automated machining operations [55]. The chips were found then to damage to the borehole surface, which clearly indicates it is necessary to make a compromise between spindle speeds and feed rates to achieve good hole quality and efficient drilling process [56]. Hence, the standard deviation of the reading is higher compared to other runs. There is a large variation between the replication due to the inconsistent hole surface roughness between the runs. Figure 10a shows the damage to a CFRP hole wall caused by aluminium chips, similar to those obtained with drill bits R8 to R13, shown in Figure 10b.

#### 3.2.1. Regression Model and Analysis of Variance (ANOVA) for the Hole Roughness

From the experimental results, a regression model was developed to estimate the hole surface roughness through all of the significant factors. The prediction model can be denoted by the following equation.

The final empirical models in terms of actual factors for hole surface roughness for CFRP (*Y*_3_),

(4) $$Y_{3}=23.46433+0.64705A-7.31683B-0.17229C-5.0139e^{-3}AC+0.05656BC$$

In the same manner, the hole surface roughness for Al7075-T6 (*Y_4_*) is given as below:

(5) $$Y_{4}=-1.12851+0.11718A-1.06063B+0.01516C+3.0493e^{-3}AB-1.0789e^{-3}AC+7.686e^{-3}BC$$

The model of hole surface roughness for the stack materials is analysed using ANOVA, as shown in Table 6 and Table 7. From the ANOVA, it is found that the two-factor interaction (2FI) is significant for both CFRP and Al7075-T6 hole surface roughness, with the *p*-value obtained less than 0.05. Moreover, the *p*-value obtained for (*Y*_3_) and (*Y*_4_) are less than 0.0001, which implies that the models are significant for CFRP and Al7075-T6 panels. For CFRP, the significant model terms affecting the surface roughness in the design space are denoted by coded factors of A, B, C, AC, and BC interactions. A further sensitivity analysis of the effect of the design factors on the accuracy of the hole surface roughness is subsequently tabulated in Table 6. From the above results, it was found that the primary clearance angle (PC: 57.56%) was the most significant contributor, followed by chisel edge angle (PC: 28.27%) and point angle (PC: 17.93%). Moreover, two input variables’ interactions, namely AC and BC, were observed in this response and it was found that the interaction of point angle with primary clearance angle (PC: 34.41%) is higher than with the chisel edge angle (PC: 15.21%). For Al7075-T6, the significant model terms are point angle (PC: 53.92%), primary clearance angle (PC:22.15%). And chisel edge angle (PC: 7.60%). Three input variables’ interactions, namely AB, AC, and BC, were observed in this response and it was found that all of these interactions modestly contributed to hole surface roughness, with PC values less than 3.5%.

For the CFRP panel, “Pred *R*^2^” of 0.6543 is not as close to the “Adj *R*^2^” of 0.8511 as one might normally expect. This may indicate a large block effect or a possible problem with the model. The model is considered to represent the fitted response values since the difference between adjusted *R*^2^ and predicted *R*^2^ did not exceed 0.2. 2FI was chosen for model fitting analysis since the 2FI model is considered as a higher degree of polynomial model when compared to linear models. “Adeq Precision” measures the signal-to-noise ratio. A ratio greater than four is desirable. The ratio of 19.7956 indicates an adequate signal. This model can be used to navigate the design space.

For Al7075-T6 panel, “Pred-*R*^2^” of 0.6341 is also not as close to the “Adj-*R*^2^” of 0.8743 as one might normally expect. This may indicate a large block effect or a possible problem within the model or data. As for the CFRP panel, the model was considered to represent the fitted response values since the difference between the adjusted *R*^2^ and predicted *R*^2^ is about 0.2. “Adeq Precision” measures the signal to noise ratio and the ratio greater than 4 is desirable. The ratio of 19.4575 indicates an adequate signal; thus, this model may also be used to navigate the design space.

**Figure 11:**
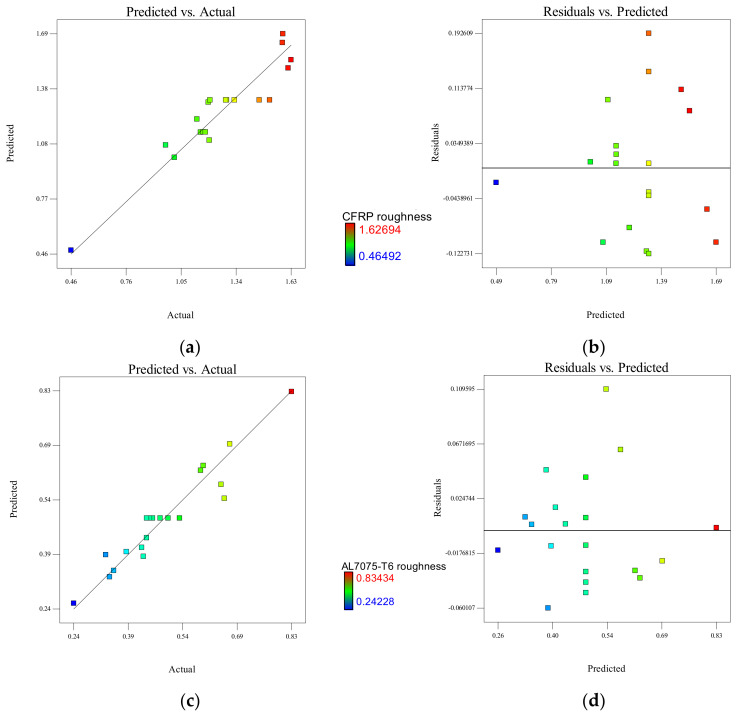
CFRP hole surface roughness analysis for (**a**) actual and predicted plot and (**b**) predicted and residual plot; Al7075-T6 hole surface roughness analysis for (**c**) actual and predicted plot and (**d**) predicted and residual plot.

The above regression models can be used to predict the values of the hole surface roughness within the limits of the factors studied. Figure 11a shows predicted versus actual plot of residual in support of the model fitness for CFRP panel. The reliability and empirical model of modification for hole surface roughness are certified when the actual value obtained through experiments is compared with the predictions of the model which is randomly scattered evenly by a 45-degree line to confirm the model fittings. The graphs show the response of the experimental results is within the range of acceptable variances when compared with the expected value of the empirical model. The SEE for the CFRP hole surface roughness is 0.0895 µm (i.e., if the predicted value is 1.0108 µm, the actual value is in the range between 0.9213 µm and 1.1003 µm). 

For the Al7075-T6 panel, the predicted and actual values are close enough and have a good agreement with the plot, almost coinciding with the 45-degree straight line, as shown in Figure 11c. The SEE for Al7075-T6 hole surface roughness is 0.0402 µm (i.e., if the predicted value is 0.5945 µm the actual value is in the range between 0.5543 µm and 0.6347 µm). For both the CFRP and Al7075-T6 panels, the RSM model can be used to predict the value since the two-thirds of the residuals data points in Figure 11b,d are shown to be within the SEE above or below the least squares line for a data set with a normal linear relationship [48].

#### 3.2.2. Effect of Geometry Parameters on Hole Surface Roughness

**Figure 12:**
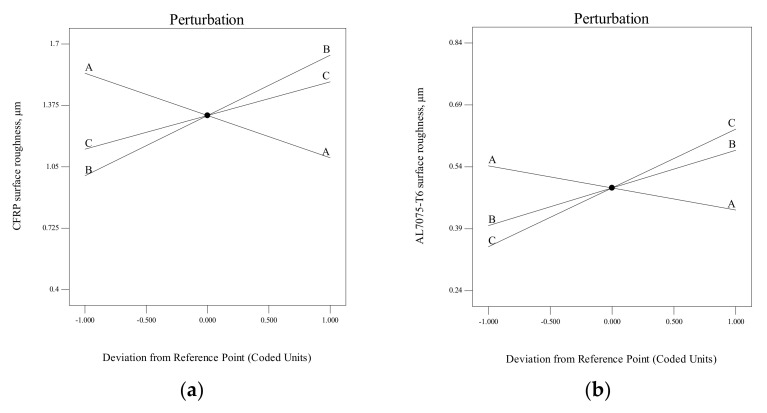
Perturbation plot for hole surface roughness: (**a**) CFRP, (**b**) Al7075-T6 (Note: A = chisel edge angle, B = primary clearance angle, C = point angle).

The sensitivity of each factor is identified through the perturbation plots presented in Figure 12 for the hole surface roughness of CFRP and Al7075-T6, respectively. The chisel edge angle (A) is mostly influenced by the hole surface roughness for both materials. When the chisel edge angle is increased from 30° (lower level −1) to 45° (higher level +1), the hole surface roughness is improved due to a greater shear angle, thus contributing to the lower chip thickness. Next, the primary clearance angle (B) crucially affects the hole surface roughness for both materials. The increase in primary clearance angle value leads to a surge in the hole surface roughness, because the weakest cutting condition is at higher primary clearance; hence, the cutting edge can easily be chipped off and obstruct the drilled hole. Lastly, the point angle (C) demonstrates a modest effect on CFRP surface roughness, and it is considered as an insignificant parameter.

**Figure 13:**
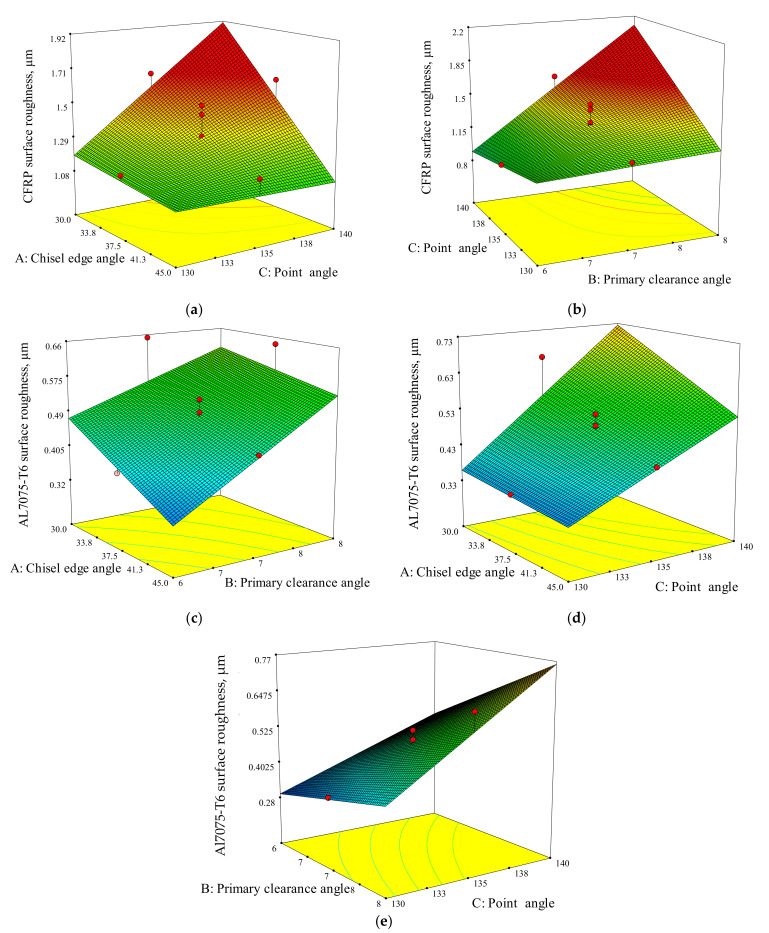
3D response surface for hole surface roughness for CFRP: (**a**) chisel edge angle and point angle, (**b**) primary clearance angle and point angle. 3D response surface for hole surface roughness for Al7075-T6: (**c**) chisel edge angle and primary clearance angle, (**d**) chisel edge angle and point angle, (**e**) primary clearance angle and point angle.

The influence of drill geometry parameters and their interaction effects can be analysed by using a 3D response graph. Figure 13a,b shows the 3D response graph of hole surface roughness for the CFRP panel; the response surface graphs are drawn by varying two parameters while maintaining the other parameters at a constant middle level. 

Figure 13a shows the response surface plot for two varying parameters, namely chisel edge angle and point angle (AC). The results show that when the point angle (C) shifts from 140° to 130° at the lowest chisel edge angle (A), the hole surface roughness of CFRP also decreases significantly. In the same way, an increase in the chisel edge angle at the highest point angle (C) generates improvement in the hole surface roughness. When the drill point angle is reduced, the cross-sectional area of undeformed chips decreased, which resulted in cutting-edge angle reduction. Hence, the thrust force is reduced, causing the cutting performance to be more efficient. This result agreed with Xu et al. [57], while drilling at a lower point angle would be able to produce a fine dust chip of composite material. The finest dust chip that can be produce by the specific bit geometry, the better the hole surface roughness that can be achieved [58].

Figure 13b shows the response surface graph for varying the two parameters of chisel edge angle and primary clearance angle (BC). It indicates that the decrease in point angle (C) at 6° of primary clearance angle (B) has an insignificant impact on the CFRP hole surface roughness. On the contrary, at the highest value of B (8°), when the point angle (C) shifts from 140° to 130°, the CFRP hole surface roughness decreases substantially. The variation in primary clearance angle (B) does not influence the hole roughness when the point angle equals 130°. On the other side, the CFRP surface roughness will be improved significantly if the primary clearance angle (B) varies from 8° to 6° at 140° of point angle.

The hole surface roughness of Al7075-T6 in the drilling of stack materials has been analysed through RSM using the generation of a 3D response surface, as illustrated in Figure 13c–e. There are three pairs of interaction that are significant to the response: chisel edge angle and primary clearance angle (AB), chisel edge angle and point angle (AC), and lastly, primary clearance angle and point angle (BC). 

From Figure 13c, observation shows that the hole surface roughness is minimum at a drill geometry of 45° of chisel edge angle and 6° if primary clearance angle. If the primary clearance angle is too high (8°), the strength of the cutting-edge of the tool is reduced; hence, the chipping process can easily occur. Therefore, the function of the drill is to be extruded more rather than cut, and the hole surface roughness increases. 

Next, Figure 13d illustrates the interaction of AC with the Al7075-T6 hole surface roughness. Regardless the value of chisel edge angle, a decrease in point angle leads to an improvement of the surface roughness. The increase in the chisel edge angle will be more beneficial on the hole surface roughness with an increase in point angle. 

Lastly, for BC interaction as shown in Figure 13e, the minimum hole surface roughness is obtained with the combination of low primary clearance angle (6°) and point angle (130°).

### 3.3. Multi-Objective Optimzation Result

Based on the development of regression function for each response correlated with the cutter geometry, the multi-objective optimisations are presented in this section. Multi-objective optimisations allow a set of optimum desired response conditions to be achieved. It can optimise all the desired response or at least keep them within the desired range. Multi-objective techniques can increase the product efficiency and aims to increase quality, cost, and time. In this study, a desirability function was applied to identify the optimised drill bit geometry. The aims of this optimisation process are to develop a tool that produces the lowest thrust force during the drilling process and minimises hole surface roughness value. Table 8 presents the goals and constraints for the factors, which aim to achieve multiple desired goals simultaneously.

Based on the goal from Table 8, one solution with its desirability levels was proposed, as tabulated in Table 9. A desirability level that is closer to 1 indicates that the goals are not easy to reach. In other words, a higher desirability index represents the closest response towards the target or ideal values. As shown in Table 9, the proposed solution gives the desirability index of 0.755.

A solution is selected based on compromises made between the responses and the constraints such as cost, efficiency, and practicality, with the real application of a drill stack material in a single-shot drilling process. In this experiment, the proposed solution for the optimised cutter geometry (a combination of 45° of chisel edge angle, 7° of primary clearance angle, and 130° of point angle) was based on the combination of the lowest thrust force measurement and the minimum hole surface roughness.

The predicted optimised result for all of the responses (*Y*_1_, *Y*_2_*, Y*_3_, and *Y*_4_) are presented in Table 10. The error between the results of the prediction and the actual experiment for proposed optimised drill bit geometry is in between 6.64% and 8.17%. The optimised value of maximum thrust force is 89.143 N and 194.623 N for CFRP and Al7075-T6, respectively. For hole surface roughness, the optimised value is 0.9995 µm and 0.2775 µm for CFRP and Al707-T6, respectively.

## 4. Conclusions

This paper presents a unique twist drill geometry that fulfil the drilling requirements of CFRP/Al7075-T6 to demonstrate improvements in minimising the thrust force and improving the hole surface roughness. Statistical analysis was used to identify the significant factors and suggest a preferable interaction of tool geometry with the objectives of reducing thrust force and hole surface roughness. In the multi-objective optimisation, thrust force and hole surface roughness for stack materials were modelled and experimentally confirmed with the effectiveness of the proposed geometry sets. These models can be used to predict the value of *Y_1_* (CFRP thrust force), *Y*_2_ (Al7075-T6 thrust force), *Y*_3_ (CFRP roughness), and *Y*_4_ (Al7075-T6 roughness) when a twist drill with different geometries is introduced. Engineers will be able to estimate the hole surface roughness value produced by the twist drill geometry and to ensure compliance with customer requirements. The major experimental findings are summarised as follows:

- The combination of the maximum primary clearance angle (PC: 58.78%) with the maximum chisel edge angle (PC: 8.87%) produces the lowest thrust force (81.16 N) in the CFRP panel while the combination of the maximum primary clearance angle (PC: 52.34%) with the maximum chisel edge angle (PC: 12.19%) produces the lowest thrust force (180.67 N) in the Al7075-T6 panel.
- A high chisel edge angle (PC: 28.27%) coupled with a low primary clearance (PC: 57.56%) and point angle (PC: 17.93%) results in the minimum hole surface roughness of the CFRP panel (0.4649 µm). For the Al7076-T6 material, the combination of a higher chisel edge angle (PC: 7.60%) with a low primary clearance angle (PC: 22.15%) and point angle (PC: 53.92%) results in the minimum hole surface roughness (0.2423 µm).
- The regression models for the responses were developed to predict the values of the results within the limits of the factors studied. The regression models are significant and can be used to predict the optimal setting since the *R*^2^ for all responses is above 80% in terms of good fit model. Furthermore, the predicted *R*^2^ is in reasonable agreement with the adjusted-*R^2^*, with a difference of less than 0.2.
- For the optimisation of drill geometry, the multi-objective optimisation method was used and the optimum value of drill geometry for a customised twist drill has been proposed. Based on goal and constraint value, one solution with its desirability index levels of 0.755 was proposed. The combination of 45° of chisel edge angle, 7° of primary clearance angle, and 130° of point angle was found to be an optimum drill geometry that would be able to drill in a single-shot process to achieve minimum thrust force and lower hole surface roughness for drilling CFRP/Al7075-T6 stacks. The solution was selected based on compromises thought thoroughly between the responses and the constraints such as quality, efficiency, and practicality for the application of aircraft assembly.
- Based on the selected optimised values of the CFRP/Al7075-T6 drill bit cutter geometry, an additional identical set of experiments was performed to validate the effectiveness of the model. From the validation results, the relative error between the predicted and actual value was calculated to be in the range of 6.76% to 8.17%, confirming the proposed optimisation.

## Figures and Tables

**Table 1:** Mechanical and physical properties of the stack materials [36].

Mechanical and Physical Properties	CFRP (Hexcel 8552)	Aluminum (Al7075-T6)
Tensile strength [MPa]	2723	558
Elasticity module [GPa]	164	71.7
Elongation [%]	1.62	13
Flexural strength [MPa]	1500	-
Interlaminar shear strength [MPa]	80	-
Density [g/cm^3^]	1.601	2.597
Thickness [mm]	3.587	3.317

**Table 2:** Chisel edge angle (A), primary clearance angle (B), and point angle (C) of the tested twist drill.

Run	A [°]	B [°]	C [°]
R1	30	6	130
R2	30	8	130
R3	30	7	135
R4	30	6	140
R5	30	8	140
R6	37.5	7	130
R7	37.5	6	135
R8	37.5	7	135
R9	37.5	7	135
R10	37.5	7	135
R11	37.5	7	135
R12	37.5	7	135
R13	37.5	7	135
R14	37.5	8	135
R15	37.5	7	140
R16	45	6	130
R17	45	8	130
R18	45	7	135
R19	45	6	140
R20	45	8	140

**Table 3:** Experimental factors with coded level (Note: A = chisel edge angle, B = primary clearance angle, C = point angle).

Input Variables	−1 (Lower Level)	Coded Level 0	+1 (Higher Level)
A [°]	30	37.5	45
B [°]	6	7	8
C [°]	130	135	140

**Table 4:** Pooled ANOVA of model for maximum thrust force for CFRP panels.

Source	Sum of Squares	df	Mean Square	F Value	*p*-Value	PC (%)	
**Model (*Y*_1_)**	2653.79	6	442.3	30.8378	<0.0001		significant
Chisel edge angle (A)	251.99	1	251.99	17.5694	0.0011	8.87%	
Primary clearance angle (B)	1669.50	1	1669.5	116.4007	<0.0001	58.78%	
Point angle (C)	48.69	1	48.69	3.3951	0.0883	1.71%	
AC	90.60	1	90.6	6.3168	0.0259	3.19%	
BC	357.09	1	357.09	24.8968	0.0002	12.57%	
B^2^	235.91	1	235.91	16.4482	0.0014	8.31%	
Residual	186.46	13	14.34			6.56%	
Lack of Fit	133	8	16.62	1.555	0.3253		not significant
Pure Error	53.46	5	10.69				
Cor Total	2840.24	19					
Std. Dev.	3.787176		*R* ^2^		0.934352		
Mean	96.36026		Adj *R*^2^		0.904053		
C.V. %	3.930226		Pred *R*^2^		0.716427		
PRESS	805.4171		Adeq Precision		18.97463		

**Table 5:** Pooled ANOVA of model for maximum thrust force for Al7075-T6 panels.

Source	Sum of Squares	df	Mean Square	F Value	*p*-Value	PC (%)	
**Model (*Y*_2_)**	7816.01	5	1563.201	22.71245	<0.0001		significant
Chisel edge angle (A)	1061.71	1	1061.707	15.42601	0.0017	12.19%	
Primary clearance angle (B)	4559.54	1	4559.542	66.24763	<0.0001	52.34%	
Point angle (C)	82.51	1	82.507	1.19878	0.2934	0.95%	
AB	1418.8	1	1418.802	20.61442	0.0006	16.29%	
BC	693.45	1	693.447	10.0754	0.0073	7.96%	
Residual	894.73	13	68.826			10.27%	
Lack of Fit	237.94	8	29.743	0.22643	0.9684		not significant
Pure Error	656.79	5	131.358				
Cor Total	8710.74	18					
Std. Dev.	8.3		*R* ^2^		0.897284		
Mean	218.9		Adj *R*^2^		0.857778		
C.V. %	3.79		Pred *R*^2^		0.787387		
PRESS	1852.01		Adeq Precision		18.86762		

**Table 6:** Pooled ANOVA of model for hole surface roughness for CFRP panels.

Source	Sum of Squares	df	Mean Square	F Value	*p*-Value	PC (%)	
**Model (*Y*_3_)**	1.2643	5	0.2529	21.58	<0.0001		significant
Chisel edge angle (A)	0.4005	1	0.4005	34.177	<0.0001	28.27%	
Primary clearance angle (B)	0.8154	1	0.8154	69.588	<0.0001	57.56%	
Point angle (C)	0.254	1	0.254	21.674	0.0005	17.93%	
AC	0.2155	1	0.2155	18.391	0.0009	15.21%	
BC	0.4875	1	0.4875	41.609	<0.0001	34.41%	
Residual	0.1523	13	0.0117			10.75%	
Lack of Fit	0.0816	8	0.0102	0.721	0.6761		not significant
Pure Error	0.0707	5	0.0141				
Cor Total	1.4166	18					
Std. Dev.	0.1082		*R* ^2^		0.8925		
Mean	1.2587		Adj *R*^2^		0.8511		
C.V. %	8.5996		Pred *R*^2^		0.6543		
PRESS	0.4898		Adeq Precision		19.7956		

**Table 7:** Pooled ANOVA of model for hole surface roughness for Al7075-T6 panels.

Source	Sum of Squares	df	Mean Square	F Value	*p*-Value	PC (%)	
**Model (*Y*_4_)**	0.3441	6	0.0574	23.0191	<0.0001		Significant
Chisel edge angle (A)	0.0286	1	0.0286	11.4813	0.0048	7.60%	
Primary clearance angle (B)	0.0834	1	0.0834	33.4749	<0.0001	22.15%	
Point angle (C)	0.203	1	0.203	81.481	<0.0001	53.92%	
AB	0.0042	1	0.0042	1.6793	0.2176	1.12%	
AC	0.0131	1	0.0131	5.256	0.0392	3.48%	
BC	0.0118	1	0.0118	4.7418	0.0485	3.13%	
Residual	0.0324	13	0.0025			8.61%	
Lack of Fit	0.0266	8	0.0033	2.8621	0.1309		not significant
Pure Error	0.0058	5	0.0012				
Cor Total	0.3765	19					
Std. Dev.	0.05		*R* ^2^		0.914		
Mean	0.489		Adj *R*^2^		0.8743		
C.V. %	10.211		Pred *R*^2^		0.6341		
PRESS	0.138		Adeq Precision		19.4575		

**Table 8:** Goals and constraints for the factors and responses.

Constraints			
Factor/Response	Goal	Lower Limit	Upper Limit
Chisel edge angle (A)	is in range	30	45
Primary clearance angle (B)	is in range	6	8
Point angle (C)	is in range	130	140
CFRP Force (F^CFRP^)	minimise	81.1563	124.557
Al7075 Force (F^Al7075^)	minimise	181.627	277.192
CFRP roughness (R_a_^CFRP^)	minimise	0.46492	1.62694
Al7075 roughness (R_a_^Al7075^)	minimise	0.24228	0.83434

**Table 9:** Proposed solution report for the optimisation process of tool geometry.

Number	A [°]	B [°]	C [°]	F^CFRP^ [N]	F^Al7075^ [N]	R_a_^CFRP^ [µm]	R_a_^Al7075^ [µm]	Desirability	
1	45.0	7	130	83.4196	211.599	1.10213	0.322027	0.755	selected
2	45.0	7	130	83.3493	211.610	1.10244	0.322686	0.755	
3	45.0	7	130	83.2189	211.630	1.10303	0.323901	0.755	
4	45.0	7	130	83.5655	211.581	1.10152	0.320739	0.755	
5	45.0	7	130	83.1027	211.649	1.10357	0.325023	0.755	
6	45.0	7	130	83.0054	211.665	1.10403	0.326037	0.754	
7	45.0	7	130	82.6354	211.731	1.10590	0.329864	0.754	

**Table 10:** Prediction of the optimised model of a twist drill bit for drilling a CFRP/Al7075-T6 stack material.

	*Y*_1_ [N]	*Y*_2_ [N]	*Y*_3_ [µm]	*Y*_4_ [µm]
Model response	95.169	210.532	1.07164	0.2586
Experimental	89.143	194.623	0.9995	0.277
Error (%)	**6.76**	**8.17**	**7.22**	**6.64**

## Data Availability

Not applicable.
